# Resonant tip-enhanced Raman scattering by CdSe nanocrystals on plasmonic substrates†

**DOI:** 10.1039/d0na00554a

**Published:** 2020-10-07

**Authors:** I. A. Milekhin, M. Rahaman, K. V. Anikin, E. E. Rodyakina, T. A. Duda, B. M. Saidzhonov, R. B. Vasiliev, V. M. Dzhagan, A. G. Milekhin, A. V. Latyshev, D. R. T. Zahn

**Affiliations:** Semiconductor Physics, Chemnitz University of Technology D-09107 Chemnitz Germany ilya.milekhin@physik.tu-chemnitz.de; Novosibirsk State University Novosibirsk Russia; A.V. Rzhanov Institute of Semiconductor Physics Novosibirsk Russia; Department of Chemistry, Moscow State University Moscow Russia; Department of Material Science, Moscow State University Moscow Russia; V.E. Lashkaryov Institute of Semiconductor Physics UA-03028 Kiev Ukraine

## Abstract

Tip-enhanced Raman scattering (TERS) has recently emerged as a powerful technique for studying the local properties of low dimensional materials. Being a plasmon driven system, a dramatic enhancement of the TERS sensitivity can be achieved by an appropriate choice of the plasmonic substrate in the so-called gap-mode configuration. Here, we investigate the phonon properties of CdSe nanocrystals (NCs) utilizing gap-mode TERS. Using the Langmuir–Blodgett technique, we homogeneously deposited submonolayers of colloidal CdSe NCs on two different nanostructured plasmonic substrates. Amplified by resonant gap-mode TERS, the scattering by the optical phonon modes of CdSe NCs is markedly enhanced making it possible to observe up to the third overtone of the LO mode reliably. The home-made plasmonic substrates and TERS tips allow the analysis of the TERS images of CdSe phonon modes with nanometer spatial resolution. The CdSe phonon scattering intensity is strongly correlated with the local electromagnetic field distribution across the plasmonic substrates.

## Abstract 2

We demonstrated resonant/non-resonant gap-mode TERS on CdSe nanocrystals analyzing of LO (up to 3rd order), SO/TO phonons, as well as Se vibrational modes.

## Graphical abstract

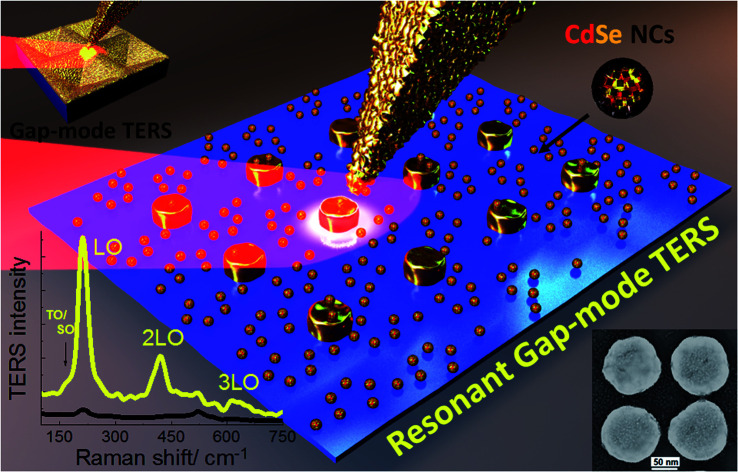

## Introduction

Technological progress in nanotechnology faces a challenge to reduce the size of active elements down to the nanoscale. Numerous parameters of the building blocks of the active nanostructured elements such as their size, shape, spatial arrangement, chemical and phase composition, impurity concentration, and mechanical strain are to be detected and controlled with high accuracy. For this purpose, non-destructive methods such as optical spectroscopy are preferable. Conventional optical methods such as vibrational Raman scattering and infrared absorption spectroscopy are, however, limited in their spatial resolution by the diffraction limit (typically in the case of Raman spectroscopy about 0.5 μm for a high-performance microscope objective). Tip-Enhanced Raman Scattering (TERS) is one of the near-field optical techniques developed to overcome the diffraction limit. The intensively developed TERS technique combines the high spatial resolution of scanning probe methods such as atomic force or scanning tunneling microscopy (AFM or STM, respectively) with the analytical capabilities of Raman spectroscopy.^1^ The strongly confined electric field of a localized surface plasmon (LSP) at the apex of a metallized AFM or STM tip enables the nanoscale analysis of nano-objects by strongly enhancing the spectroscopic signal in the vicinity of the tip. The local phonon properties can be studied by analyzing the TERS maps acquired *via* scanning the plasmon-active tip across the sample surface under laser illumination.^1^ The significant near-field enhancement stems from the 4^th^ order dependence of the Raman enhancement on the local electric field magnitude (EF ∼ *E*^4^).^2^

The TERS enhancement reaches values up to 10^7^ for various organic materials, nanocarbons including carbon nanotubes,^3–5^ one-dimensional carbyne,^5^ graphene,^5,6^ and other 2D materials,^7,8^ while the spatial resolution steadily improves from tens down to sub-nanometers.^9–12^ The recent advances in TERS imaging are discussed in several comprehensive reviews.^9,11–13^

However, compared to the abovementioned materials, near field optical characterization of inorganic crystalline semiconductor nanostructures has been rarely reported so far. Only in 2005, the TERS enhancement by optical phonons in a 10 nm CdS film using a sharp metal tip with an apex radius of 25 nm was demonstrated,^14^ without specifying the actual spatial resolution. Later, the phonon confinement effect and contributions to the Raman phonon scattering of crystalline and amorphous phases in single Ge nanowires with a diameter of less than 20 nm were studied,^15^ while variations at the nanoscale in the local strain and composition of Ge/Si nanowires were determined in ref. 16. The capability to probe the crystal symmetry using TERS selection rules in BaTiO_3_ and LiNbO_3_ nanostructures was also demonstrated.^17,18^

Each of the two approaches to the instrumental implementation of TERS, namely STM-TERS or AFM-TERS configurations, has its own advantages and drawbacks. The STM-TERS experiments require special experimental conditions such as well-defined conductive substrates, which are free of carbon contamination, fabrication of ultra-sharp metal STM tips, nitrogen atmosphere^19^ or ultra-high vacuum conditions^20^ needed for cryogenic temperatures to be achieved.^21^ AFM-TERS, on the other hand, can be realized under ambient conditions or even in a liquid environment.^22,23^ Note that, under ambient conditions a thin water layer is formed on the surface of most hydrophilic samples. It may play an important role in the surface diffusion of analyte molecules or cause undesirable effects such as the transition of molecules to the tip or molecule decomposition, leading to contamination of the tip. In addition, low-cost home-made TERS tips fabricated using a chemical etching of metal microwires,^24^ a direct vacuum deposition of a metal on the surface of standard AFM tips or functionalization of a metallic tip with organic molecules^25^ can be effectively used for different analytical tasks.

A dramatic increase of the TERS enhancement and a better lateral resolution, both resulting in higher contrast TERS imaging, can be achieved in the so-called gap-mode TERS.^24,25,27^ In this mode, the organic or inorganic nanostructures are placed on a flat or nanostructured metallic substrate in the gap between the sharp AFM tip and the metal substrate.^12^ The gap mode is especially efficient when a nanostructured metal substrate is used, providing an extremely high local field enhancement and its spatial confinement.^28^ The gap-mode geometry allowed unique TERS experiments with single molecules to be performed including DNA imaging and sequencing at room temperature with subnanometer resolution.^29^ STM-TERS imaging of a single H_2_TBPP molecule on a Ag (111) surface with spatial resolution below one nanometer resolved the inner structure of the molecule at about 80 K under ultrahigh-vacuum conditions.^30^ Superior TERS images of a single CoTPP molecule on a Cu (100) surface with subnanometer-scale resolution resolving the patterns of normal modes were also obtained using an ultrahigh-vacuum STM setup at 6 K.^21^

For inorganic nanostructures, significant progress has been achieved for the local gap-mode TERS analysis of 2D semiconductors.^31^ Recently we employed gap-mode AFM-TERS for determining the local strain in mechanically transferred monolayers of MoS_2_ on Au nanostructures.^8,26,32^ The biaxial strain in an ultrathin MoS_2_ layer induces a shift of the E_2g_ phonon mode, which is probed by TERS spectroscopy with a spatial resolution of less than 25 nm.^8^ A dramatic Raman enhancement factor of 5.6 × 10^8^ accompanied by a high spatial resolution of 2.3 nm was achieved in gap-mode TERS for monolayer MoS_2_ on Au nanodisks and allowed us to observe, for instance, the MoS_2_ phase transition from 2H to 1T during TERS mapping.^26^ Recently, we observed the TERS enhancement due to optical phonon modes in CdSe NCs as well as in a CoPc thin film deposited on an Au nanodisk array.^33,34^

In this paper, we extend our investigations by demonstrating gap-mode AFM-TERS imaging for the local analysis at the nanometer scale of submonolayers of colloidal CdSe nanocrystals deposited on home-made plasmonic substrates consisting of an array of Au nanodisks and for comparison on a commercial SERS substrate.

## Methods

### Sample fabrication

**Fig. 1:**
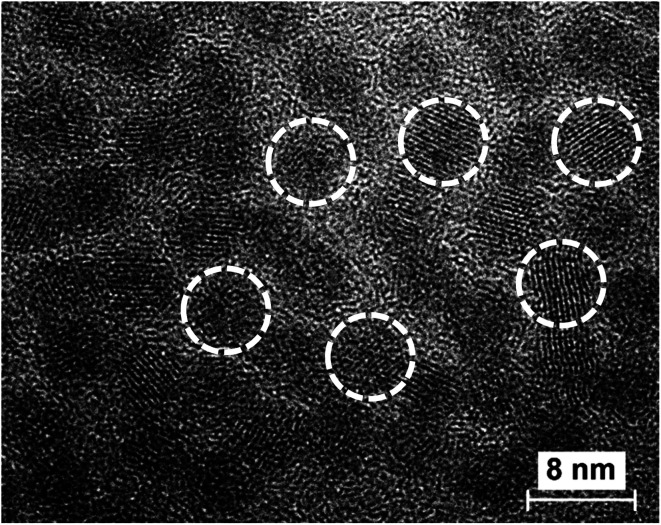
TEM image of colloidal CdSe NCs. The white circles indicate single CdSe NCs for clarity.

CdSe nanocrystals (NCs) were synthesized by colloidal chemistry following a procedure reported previously^35^ using oleic acid as a stabilizer. Using transmission electron microscopy (TEM), we found that CdSe NCs predominantly have a zinc-blende crystalline structure and an average size of 5–6 nm (Fig. 1).

Colloidal CdSe NCs in a toluene solution with a concentration of 10^−3^ M mixed together with a solution of behenic acid in molar ratios of 1–3 : 1 were used for homogeneous transfer onto the nanostructured plasmonic substrates by means of a modified Langmuir–Blodgett (LB) technique as described in ref. 36. The structures were further annealed at 150 °C to remove the organic molecules. As a result, submonolayers of colloidal CdSe NCs were formed on Au nanodisk arrays and Klarite plasmonic substrates.

**Fig. 2:**
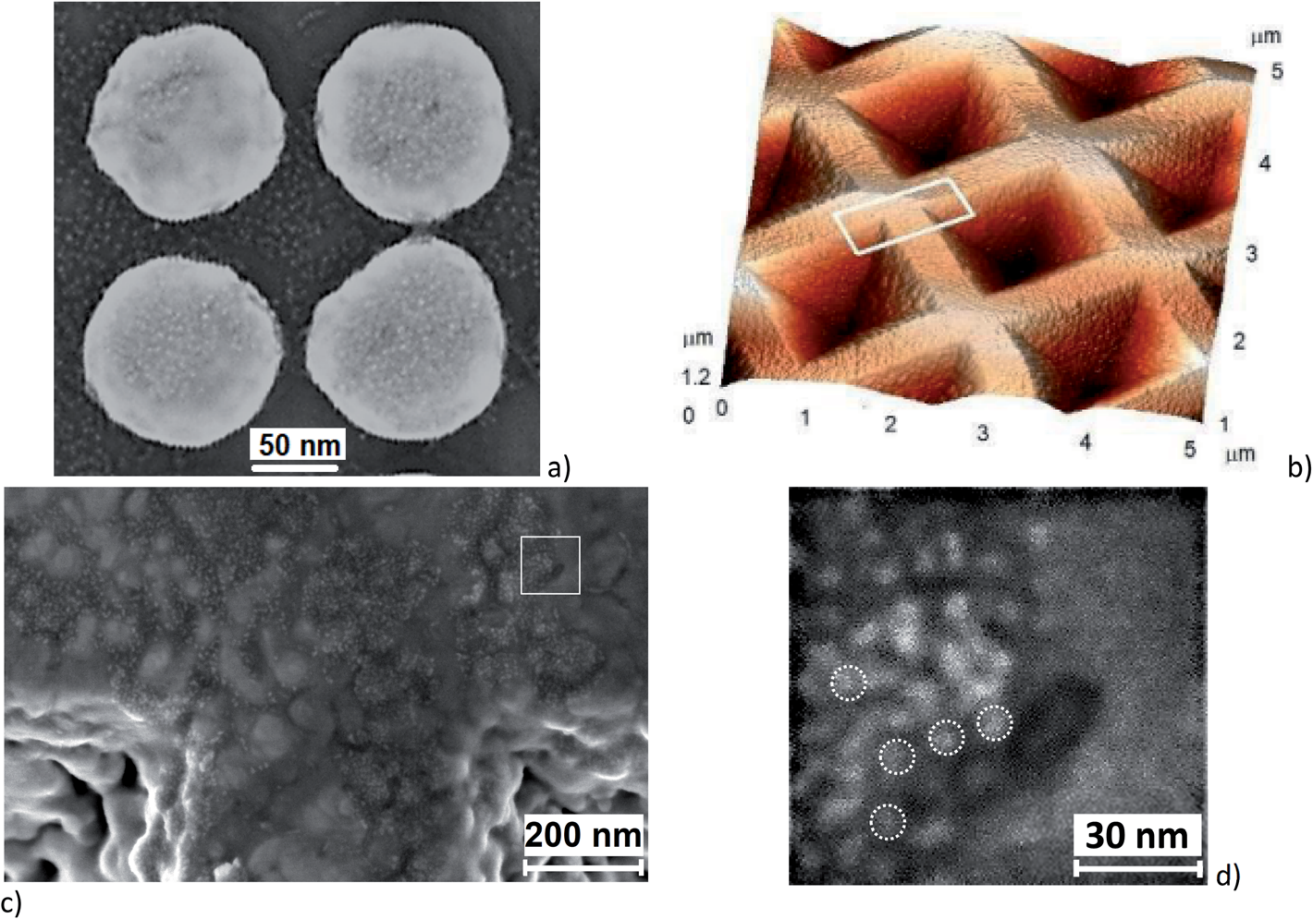
(a) SEM image of a fragment of the Au nanodisk array (four large bright spots) covered with CdSe NCs (small bright spots). (b) AFM image of a Klarite® structure with a submonolayer coverage of CdSe NCs. CdSe NCs are not clearly resolved in the image. The white rectangle indicates the area shown in the SEM image in (c). (d) A fragment of the SEM image, marked by the white rectangular in (c), showing submonolayer coverage of the surface by CdSe NCs.

The arrays of Au nanodisks with a size of 80–130 nm and a height of 50 nm were fabricated using electron beam lithography (Raith-150, Germany) on a Si (001) substrate covered with an 8 nm thick SiO_2_ layer as described in ref. 37. The period of the array was 200 nm. Fig. 2a shows a typical SEM image of Au nanodisks with a uniform CdSe NC coverage over the whole substrate. The fabricated nanodisks in each array have the same size with an accuracy of about ±5 nm.

A typical AFM image of a Klarite® substrate representing an array of inverted Si pyramids covered by Au nanoclusters^13^ is shown in Fig. 2b. The base of each pyramid is a square with a size of 1.5 × 1.5 μm^2^, while the pit depth is 1 μm. Au nanoclusters having an irregular shape and a typical size of 50–100 nm form a continuous rough Au layer. The inverted pyramids serve as resonators, in which the electromagnetic field is enhanced. The SEM image of the rectangle area marked in Fig. 2b is presented in Fig. 2c, while Fig. 2d is a more detailed view of the square in Fig. 2c. As one can see from Fig. 2c and d, CdSe NCs are predominantly deposited in the grooves between Au nanoclusters forming a random network on the rough Au surface.

### TERS tip fabrication

**Fig. 3:**
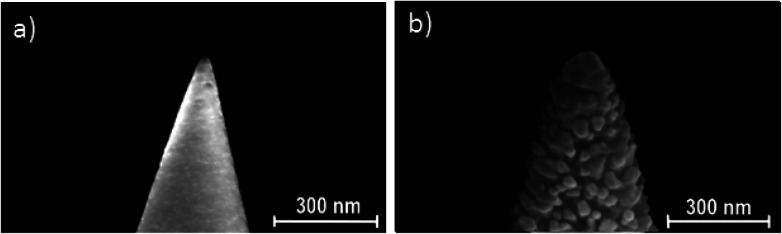
(a) SEM image of the apex of the as-purchased Si AFM tip (a). (b) SEM image of the AFM tip apex covered with Au (TERS probe).

Home-made AFM tips with single Au nanoclusters at the tip apex were used for reproducible TERS experiments. To fabricate these TERS-active tips, we used standard AFM cantilevers purchased from Tipsnano (https://tipsnano.com/) with a specified tip radius of 10 nm as shown in Fig. 3a. A gold layer with a nominal thickness of 200 nm was thermally evaporated onto the surface of the silicon AFM cantilevers by placing the tip axis parallel to the evaporation beam. In order to improve Au adhesion to silicon, 5 nm of titanium was deposited prior to Au evaporation. The morphology of the Si AFM tip apex was studied by SEM before (Fig. 3a) and after (Fig. 3b) Au deposition. As a result of the gold evaporation, polycrystalline Au grains appear on the Si tip surface. As can be seen in Fig. 3b, a single gold cluster is reproducibly formed at the tip apex with a typical size of 80–90 nm. Most probably, this single cluster acts as the main LSP source for the TERS excitation in our experiments. An argument strongly corroborating this assumption is the high spatial resolution of TERS images obtained with such tips.

### TERS experiment

**Fig. 4:**
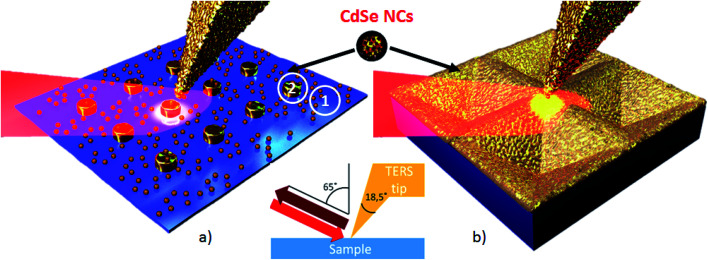
Scheme of the TERS experiment for CdSe NCs deposited on an Au nanodisk array (a) and a Klarite substrate (b). Points 1 and 2 in (a) mark typical positions for collecting conventional and gap-mode TERS spectra, respectively. In (b) CdSe NCs are hardly visible due to the small scale of the sketch.

A Horiba NanoRaman platform consisting of an AIST-NT AFM coupled to a XploRA Raman spectrometer was used for the TERS experiments in the side illumination geometry under ambient conditions. P-polarized laser excitation and collection of the (unpolarized) backscattered light were realized in this geometry through a long working distance objective (100×, 0.7 NA). The 638.2 nm (1.94 eV) and 785.3 nm (1.58 eV) lines of solid-state lasers with a power of 1 and 3 mW, respectively, were used for excitation. Such laser power induces no noticeable sample damage and reveals reproducible TERS. The experimental geometry on the samples is shown schematically in Fig. 4a and b. The angle of incidence/collection of light is 65° with respect to the surface normal. This is a very efficient geometry for exciting hybrid plasmon states (so-called gap plasmon) in both the Au nanodisk and the TERS tip. Due to the inclined laser beam, the laser spot on the surface is ellipsoidal and the illuminated area is enlarged to approximately 1.3 μm^2^ while minor and major ellipsoidal axes are equal to 1 and 1.7 μm, respectively. The collected light was dispersed by a 600 lines per mm grating on an electron-multiplying charge-coupled device (EMCCD) with a resulting spectral resolution of around 12 and 9 cm^−1^ for 638.2 nm and 785.3 nm lasers, respectively. The acquisition time for a single spectrum and the step size during TERS mapping were 0.3 s and 6 nm, respectively. Colloidal CdSe NCs deposited on the substrate are randomly distributed in the position between the plasmon substrate and TERS tip. The NCs are located in the field of the gap plasmon that corresponds to the gap-mode TERS geometry and experience strong enhancement. The semicontact AFM mode was employed in the measurements.

## Results and discussion

Conventional and gap-mode TERS spectra of CdSe NCs deposited on Au nanodisk arrays were collected on a bare Si/SiO_2_ area between Au nanodisks (area 1 in Fig. 4a) and on nanodisks (area 2 in Fig. 4a). The optical band gap of our CdSe NCs, corresponding to their size of 5–6 nm, is about (2.05 ± 0.05) eV (610 nm).^38^ Therefore, resonant Raman scattering from CdSe NCs is expected upon excitation in the red spectral region.

**Fig. 5:**
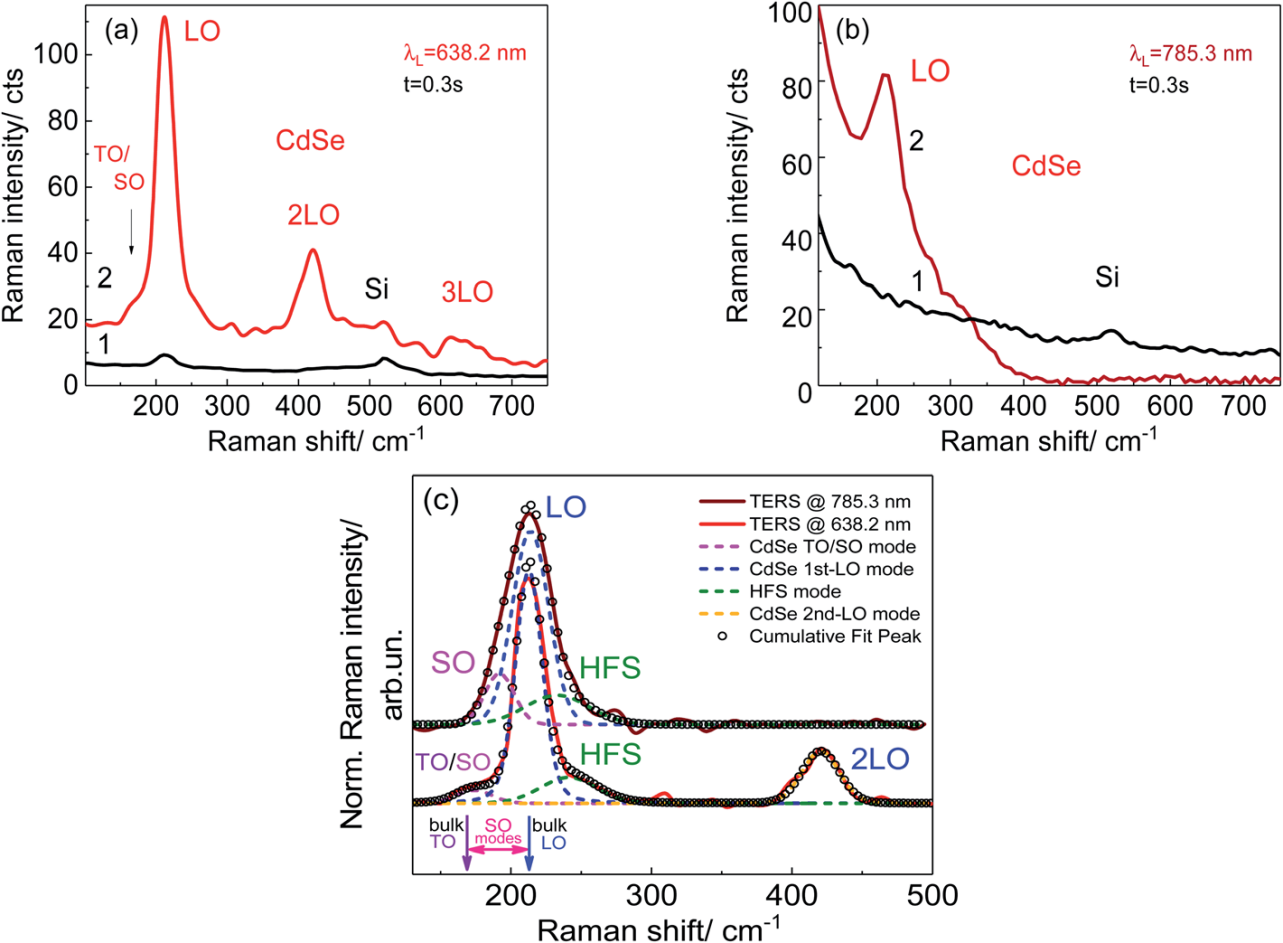
(a) Comparison of conventional TERS and gap mode TERS spectra of CdSe NCs recorded on a Si/SiO_2_ substrate away from the nanodisks (curve 1, area 1 in Fig. 4a) and at the edges of Au nanodisks (curve 2, area 2 in Fig. 4a). Nanodisk size of 80 nm was used in the experiment. (b) Gap mode TERS spectra of CdSe NCs on a flat gold area (curve 1) and at the vertices of the inverted pyramids (curve 2) of the Klarite substrate. (c) Comparison of normalized TERS spectra recorded for CdSe NCs on the Au nanodisk array and the Klarite substrate excited with 638.2 and 785.3 nm, correspondingly. Here, the background seen in (a) and (b) was subtracted for the convenience of comparison between the two spectra and curve fitting.

A comparison of conventional and gap-mode TERS spectra measured with 638.2 nm laser excitation is shown in Fig. 5a (curves 1 and 2, respectively). The conventional TERS spectrum (curve 1) reveals weak features at 212 and 520.5 cm^−1^, attributed to Raman scattering by longitudinal optical (LO) phonons from CdSe NCs^39^ and the Si substrate,^40^ respectively. The gap-mode TERS spectrum of CdSe NCs recorded on the same sample (curve 2) differs drastically. The intensity of the LO phonon mode from CdSe NCs deposited on the nanodisk plasmonic structure increases by a factor of about 25 (which determines the ratio of the gap-mode TERS and conventional TERS signals, see Fig. 5a spectra 2 and 1 respectively). In addition, weaker modes at double and triple LO frequencies are also observed, corresponding to the first (2LO) and the second (3LO) overtones of the Raman phonon scattering process.^39^ Registering overtones indicate the resonant nature of the Raman scattering process at 632.8 nm excitation. In our case, the conditions for resonant TERS are fulfilled indeed – the laser excitation energy coincides both with the energy of the gap plasmon and with the bandgap energy of CdSe NCs. The frequency position of the LO phonon, around 212 cm^−1^, corresponds well to its value in CdSe bulk or in large-sized CdSe NCs, for which the phonon confinement effect is not significant.^39,41,42^ Even though a 2–3 cm^−1^ lower frequency position could be expected for 5–6 nm CdSe NCs,^43^ the real particle size in our case could be larger due to partial merging of individual NCs in the course of the annealing and consequent removal of the ligand spacer between them.

In the case of the Klarite substrate, no Raman signal of CdSe NCs could be detected with *λ*_exc_ = 638.2 nm (spectrum not shown). Changing the excitation to 785.3 nm leads to an observable TERS spectrum (Fig. 5b, curve 2) of intensity comparable to that at 638.2 nm excitation on the Au nanodisk substrate, with no 2LO and 3LO features being observed. The intensity of the LO phonon mode from CdSe NCs deposited on the Klarite plasmonic structure increases by an estimated factor of about 15 (determined as the signal ratio between the gap-mode TERS acquired inside an inverted pyramid and noise level of the spectrum near 210 cm^−1^ measured at the flat gold in between inverted pyramids of the Klarite substrate, see Fig. 5b spectra 2 and 1 respectively). This observation can be explained by the non-resonant nature of the Raman scattering process in the case of *λ*_exc_ = 785.3 nm, because the latter is below the bandgap even for bulk CdSe (around 720 nm (ref. 44)). This observation is also in agreement with observing no overtones under similar excitation conditions (*λ*_exc_ = 785.3 nm) in SERS of CdSe NCs with a 6 nm diameter and an emission peak at 640 nm by Hugall *et al.*^45^ Note that no TERS signal from optical phonons in CdSe NCs on the flat gold was detected (Fig. 5b). A comparison of normalized TERS spectra recorded for CdSe NCs on the Au nanodisk array and the Klarite substrate excited with 638.2 and 785.3 nm, respectively, is shown in Fig. 5c along with the peak fitting. In these spectra, the background seen in Fig. 5a and b was subtracted for the convenience of comparison of the two spectra and their deconvolution.

A remarkable spectral difference of the present TERS spectra of CdSe NCs on the two substrates is the LO mode line shape at 212 cm^−1^. While it is narrow accompanied by high and low-frequency shoulders seen clearly at *λ*_exc_ = 638.2 nm on the nanodisk substrate, a single broader feature is observed at *λ*_exc_ = 785.3 nm for the Klarite substrate. Fitting both Raman spectra in the region between 150 and 250 cm^−1^ by three Gaussians allowed us to determine the frequency positions and the spectral widths of the deconvoluted modes. As one can see from Fig. 5c both spectra can be fairly well fitted by three components.

The shoulder on the lower-frequency side of the LO peak can be attributed to the combination of transverse optical (TO)/surface optical (SO) modes. The SO modes are expected in the frequency range between the bulk TO and LO frequencies (Fig. 5c).^46,47^ The low signal of both TO and SO modes together with the low spectral resolution in our TERS experiments does not allow these modes to be resolved separately. However, observing the SO mode may be related to the noticeable contribution of longitudinal atomic motion to the surface atoms. According to earlier theoretical prediction,^46,47^ all strongly localized optical vibrational modes in spherical NCs may have mixed TO–LO nature, with different ratios of the three contributions for each of the NC modes. As to the TERS spectrum at *λ*_exc_ = 785.3 nm, the SO mode can only be revealed as a result of the fitting procedure while the TO mode is apparently suppressed under non-resonant conditions.^48^

As to the assignment of the high-frequency shoulder (HFS), several possible origins were discussed so far,^39^ such as multiphonon processes involving both optical and acoustical phonons,^49–51^ manifestation of the phonon density of states in NCs,^52–54^ and vibrational modes of selenium on the surface of CdSe NCs.^34^ In the case of TERS spectra of CdSe NCs, the assignment of this spectral feature to the vibrations of surface selenium, which has modes in the range of 230–250 cm^−1^,^55^ was favored.^34^ Therefore, it is reasonable to assume that the vibrations of the atoms on the NC surface are preferably enhanced in the TERS experiment, compared to the vibrations in the inner part of the NC. Furthermore, the magnitude of the enhancement of surface Se-related Raman scattering can be expected to be more or less constant for a given system. It is determined mainly by the number of Se–Se bonds on the surface of CdSe NCs and the proximity of the NC surface to the plasmonic metal surface.

Note that the frequency positions of the most pronounced LO mode in both Raman spectra coincide, while its spectral width in the spectrum at *λ*_exc_ = 785.3 nm (33 cm^−1^) is significantly larger compared to that observed at *λ*_exc_ = 638.2 nm (23 cm^−1^). The enhancement of the LO phonon scattering of the CdSe NCs is expected to be governed by the resonance conditions, because of the well-known strong electron–LO phonon coupling in this compound, especially under resonant excitation.^56^ Therefore, the much stronger enhancement of the LO peak intensity in the present work, as compared to our previous study,^34^ probably can be explained by matching the resonance conditions of *λ*_exc_ = 638.2 nm with the bandgap of the CdSe NCs, corroborated by a larger 2LO/LO intensity ratio in the present study. It should be noted that in a previous study^34^ the distance between nanodisks was 150 nm. However, in accordance with ref. 59, Au nanodisk arrays with a disk size of 80 nm and pitches of 130, 150, 200, and 250 nm reveal the same LSPR wavelength which amounts to (640 ± 5) nm. Because of the invariability of LSPR energy for the arrays with Au nanodisks with the same disk size and different pitches, the nanodisks can be considered as non-interactive. The relatively narrow spectral width of the LO mode at *λ*_exc_ = 638.2 nm can be also explained by the influence of resonant conditions, which may promote the selective enhancement of Raman scattering from the NCs having their optical bandgap energy closest to that of laser excitation.^57^ Indeed, in an ensemble of CdSe NCs, individual NCs may have slightly different NC sizes and shapes, and some NCs can agglomerate thus causing not only variation of bandgap energy but also phonon mode broadening. Under resonant conditions, NCs with a narrow size and shape distribution, the bandgap energy of which coincides with that of the laser excitation, predominantly contribute to the Raman scattering process.

As far as the spectrum at *λ*_exc_ = 785.3 nm (Fig. 5c) is concerned, both low- and high-frequency shoulders of the LO band, corresponding to SO and Se vibrational modes, respectively, are hidden by the broader LO band (compared to the 638.2 nm spectrum) and can be resolved only by the curve fitting procedure (Fig. 5c).

**Fig. 6:**
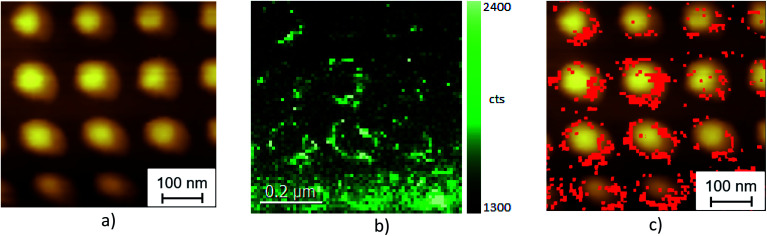
(a) AFM topology image of the Au nanodisk array with CdSe NCs. (b) TERS intensity image of LO phonon mode from CdSe NCs, obtained simultaneously with the AFM image shown in (a). (c) Superposition of the AFM image and TERS intensity from the same sample area indicating that the strong TERS signal originates predominantly from the Au nanodisk edges. (c) is created by overlaying the background subtracted TERS map (b) on top of the AFM topography (a).

In order to get a better understanding of TERS by CdSe NCs on plasmonic substrates, the AFM morphology map was acquired simultaneously with the TERS map. TERS images of CdSe NCs on the Au nanodisk array were obtained for an array period of 200 nm and a size of Au nanodisks of 80 nm as determined from AFM (Fig. 6a) and SEM (see ESI Fig. SI-4†). The acquired TERS image is shown in Fig. 6b. The TERS image was created by acquiring the Raman intensity in the range of 200 to 230 cm^−1^ in the CdSe LO mode at 212 cm^−1^. As can be seen from Fig. 6b, the TERS image represents an array of rings with their diameter and periodicity matching the Au disks observed in the AFM image. An overlay of TERS and AFM maps clearly shows that the strongest TERS signal originates from the edges of Au nanodisks (Fig. 6c). This is in accordance with our finite element method calculation of the electric field distribution in the vicinity of an Au nanodisk, which shows the maximal field enhancement near its edges.^32^ Since the TERS enhancement is proportional to the 4^th^ order of the local electric field magnitude (EF ∼ *E*^4^),^2^ the TERS images deliver direct information on the EM field distribution across the nanodisk array.

Note that similar round shape TERS images were experimentally observed for MoS_2_ monolayers deposited on Au nanodisk arrays^26^ again indicating that strong TERS signals are observed at the edges of the nanodisks due to the high electric field enhancement occurring there.

**Fig. 7:**
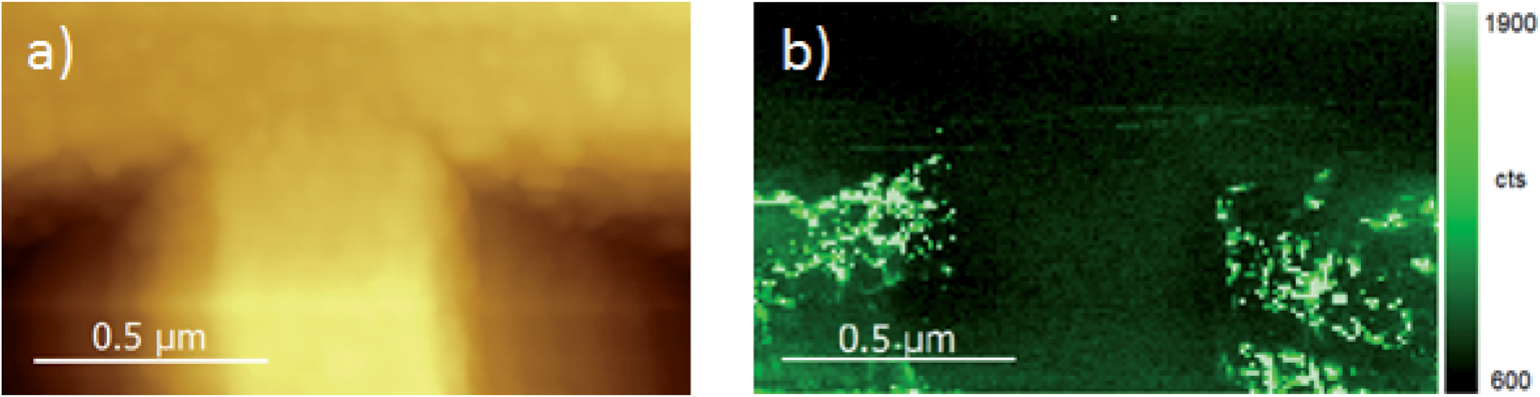
(a) AFM image of vertices of two inverted pyramids of a Klarite structure. (b) TERS map of LO CdSe phonon mode obtained from the same region shown in (a).

The region chosen for the TERS mapping on the Klarite substrate includes two vertices of the inverted pyramids as shown in Fig. 2b. Fig. 6a and b display the AFM and TERS maps of CdSe NCs on the Klarite substrate obtained simultaneously. As can be seen from the comparison of the AFM (Fig. 7a) and TERS (Fig. 7b) images of this area, the TERS signal originates from the vertices of the inverted pyramids, while NCs located on the surface of flat Au areas reveal featureless spectra in the spectral region of optical phonons in CdSe NCs (Fig. 5b, curve 1). This observation is in accordance with the results of calculations of the electromagnetic field distribution on Klarite substrates at 785.3 nm excitation,^58^ which predicted the maxima of the field intensity near the vertices of the pyramids. Therefore, TERS images allow the local EM field distribution to be probed for both Klarite and nanodisk array structures.

The comparative TERS study of CdSe NCs deposited on plasmonic substrates with different surface morphologies allows several TERS geometries to be probed. First, the conditions for resonant gap-mode TERS by optical phonons in CdSe NCs are fulfilled for the NCs placed in the gap between the TERS tip and an Au nanodisk since the coincidence of excitation energy, interband electronic transition energy in CdSe NCs, and LSPR energy in Au nanodisks is achieved. This mode provides maximal TERS enhancement for the NC phonon modes. Secondly, near-resonant TERS represents an effective tool for local Raman analysis of nanomaterials, the phonon response of which is additionally enhanced by the local electric field induced in the optical cavity of a Klarite plasmonic substrate. Finally, non-resonant conventional TERS and gap-mode geometries, for which the energies mentioned above are detuned, stress the importance of an appropriate choice of experimental conditions for an ultimate TERS enhancement.

## Conclusions

In this paper, we report the TERS mapping of submonolayers of CdSe NCs deposited on different plasmonic substrates. In the case of Au nanodisks, we observed a ring-like shape in the TERS image originating from the enhancement of the Raman modes of CdSe NCs matching the rim of the disks, while a strong TERS enhancement was evidenced at the vertices of the inverted pyramids in the case of the Klarite substrate. The strong TERS signal originates from the areas on the plasmonic substrates, in which the local electromagnetic field is strongly enhanced, as confirmed by calculations.^58,59^ The TERS images obtained using 638.2 nm excitation on Au nanodisks showed resonant TERS since the energy of the incident laser light coincides with that of the gap-plasmon energy of the metal nanostructures and is close to the interband electronic transition energy in CdSe NCs. However, the inverted pyramids of the Klarite substrate showed TERS located at the corners only for the 785.3 nm excitation. The TERS mapping of CdSe NC submonolayers on plasmonic substrates indicates that the metal nanostructure morphologies define the gap plasmon energy and resonant TERS enhancement. We show that the fulfilment of the gap-plasmon conditions allows a local TERS analysis of LO and SO/TO phonons, as well as Se vibrational modes, in CdSe NCs on Au nanodisk arrays to be performed. Resonant gap-mode TERS demonstrates a dramatic increase in the intensities of the NC phonon modes in comparison with non-resonant gap-mode TERS and conventional TERS. The intensity increase of the LO phonon peak is accompanied by narrowing of the phonon mode most probably due to size-selective Raman scattering.

## Authors' contribution

IM, MR, and KA performed experiments. ER, TD, BS, and RV contributed to the sample preparation. IM, MR VD, AM, AL, and DRTZ contributed to planning and understanding the experiments and results. IM wrote the manuscript and all other authors corrected and agreed to the manuscript.

## Conflicts of interest

There are no conflicts to declare.

## Supplementary Material

NA-002-D0NA00554A-s001
