# Supplementary material for: Resonant tip-enhanced Raman scattering by CdSe nanocrystals on plasmonic substrates
Source: Nanoscale Adv. 2020 Oct 7;2(11):5441–9. doi: 10.1039/d0na00554a (PMC9417628; doi:10.1039/d0na00554a)
Supplement: NA-002-D0NA00554A-s001 [file NA-002-D0NA00554A-s001.pdf]

Electronic Supplementary Material (ESI) for Nanoscale Advances.
This journal is © The Royal Society of Chemistry 2020

Supplementary Information

Resonant Tip-enhanced Raman Scattering by CdSe Nanocrystals on Plasmonic Substrates

I.A. Milekhin[a], M. Rahaman[a], K.V. Anikin[c], E.E. Rodyakina[b,c], T.A. Duda[c], B.M. Saidzhonov[d,e], R.B. Vasiliev[d,e], V.M. Dzhagan[f], A.G. Milekhin[b,c], A.V. Latyshev[b,c], D.R.T. Zahn[a]

[a] Semiconductor Physics, Chemnitz University of Technology, D-09107 Chemnitz, Germany
[b] Novosibirsk State University, Novosibirsk, Russia
[c] A.V. Rzhanov Institute of Semiconductor Physics, Novosibirsk, Russia
[d] Department of Chemistry, Moscow State University, Moscow, Russia
[e] Department of Material Science, Moscow State University, Moscow, Russia
[f] V.E. Lashkaryov Institute of Semiconductor Physics, UA-03028 Kiev, Ukraine

ilya.milekhin@physik.tu-chemnitz.de

SI-1: Reproducibility of Gap-mode TERS spectra

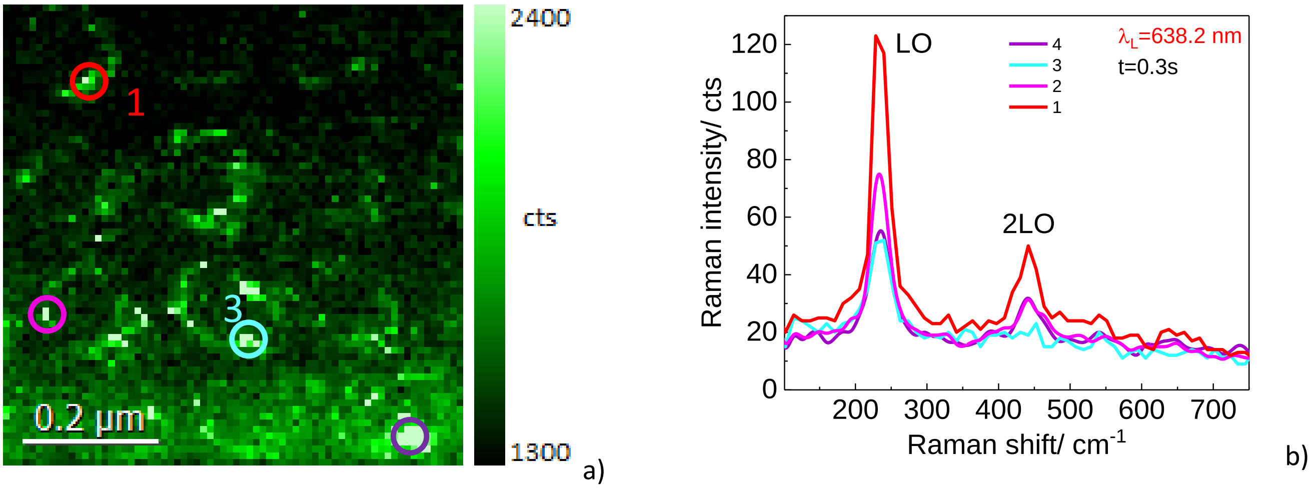

Fig. SI1. (a) TERS intensity image of LO phonon mode from CdSe NCs. (b) Comparison of different TERS spectra of CdSe NCs measured in gap mode. Colored circles in (a) indicate regions where TERS spectra were taken for comparison.

SI-2: Determination of spatial resolution of TERS mapping

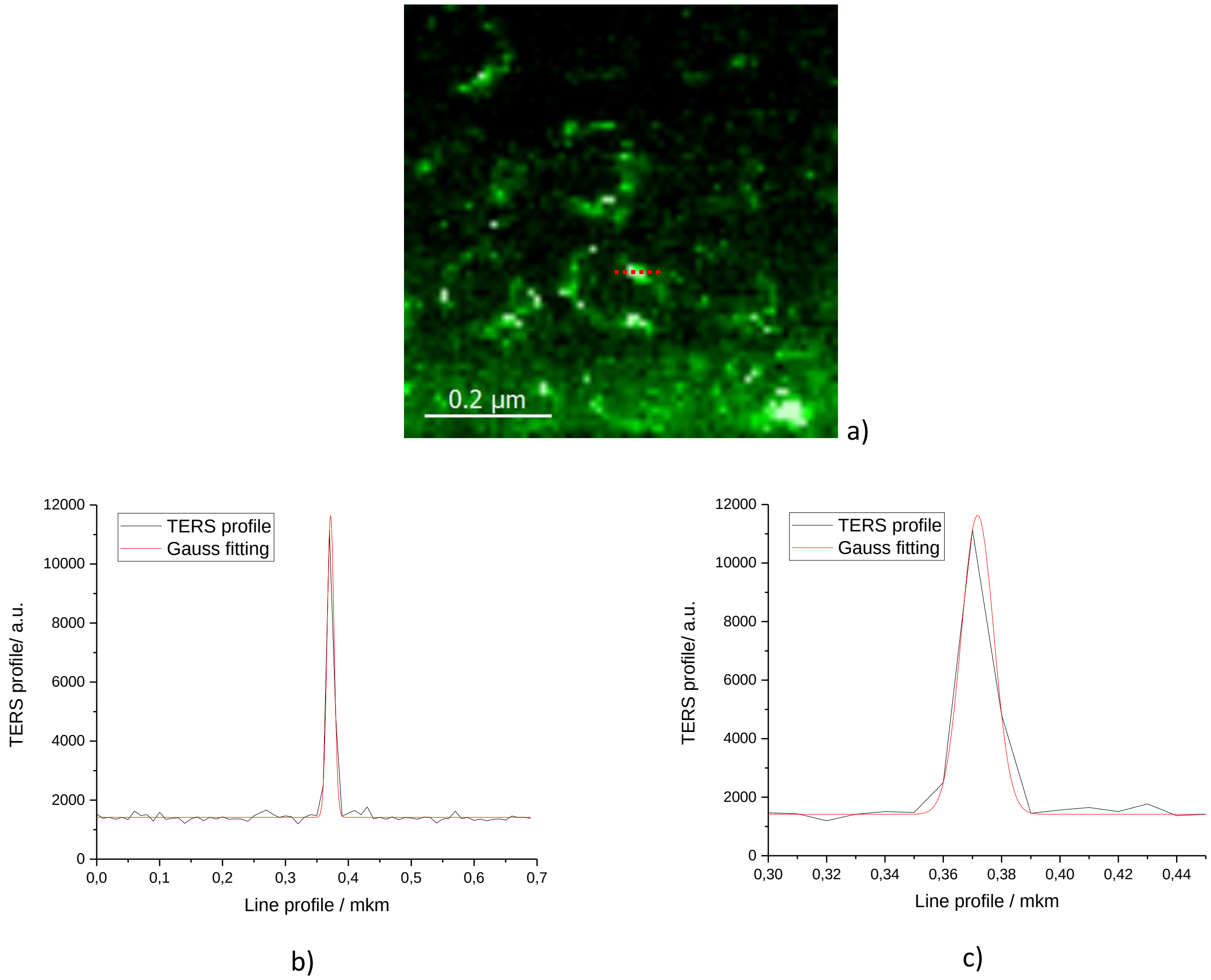

Fig. SI2. (a) TERS intensity image of LO phonon mode from CdSe NCs with a red line represents line profile. (b) The line profile was taken in accordance with a red line drawn at (a). (c) Zoomed region from (b) with a Gauss fitting for determination of spatial resolution for TERS mapping.

SI-3: Determination of spatial resolution of TERS mapping

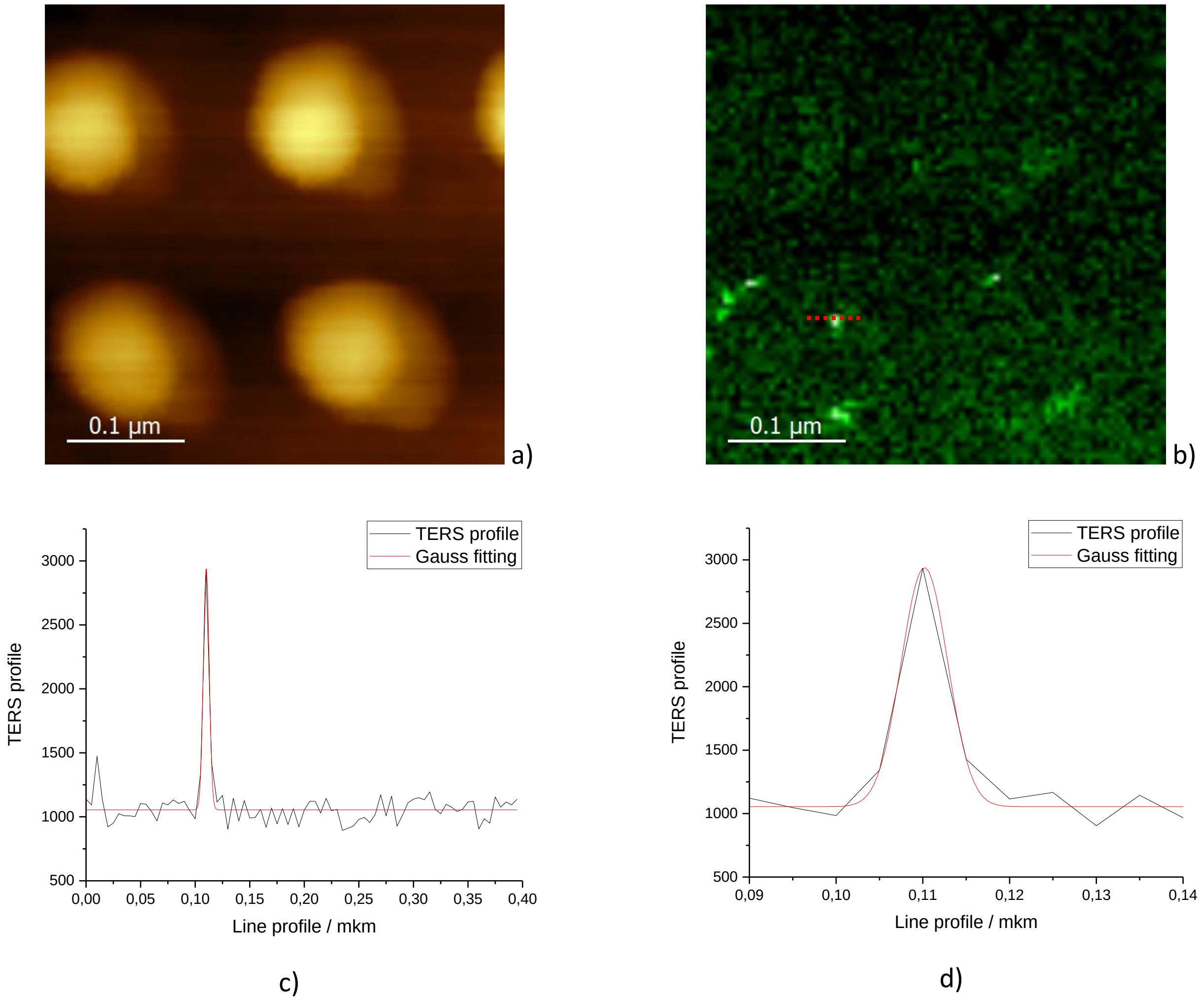

Fig. SI3. (a) AFM topology image of Au nanodisk array with CdSe NCs. (b) TERS intensity image of LO phonon mode from CdSe NCs measured simultaneously with the AFM image shown in figure (a). (c) Line profile was taken in accordance with a red line drawn at (b). (d) Zoomed region from (c) with a Gauss fitting for determination of spatial resolution for TERS mapping.

SI-4: SEM image of nanodisk array

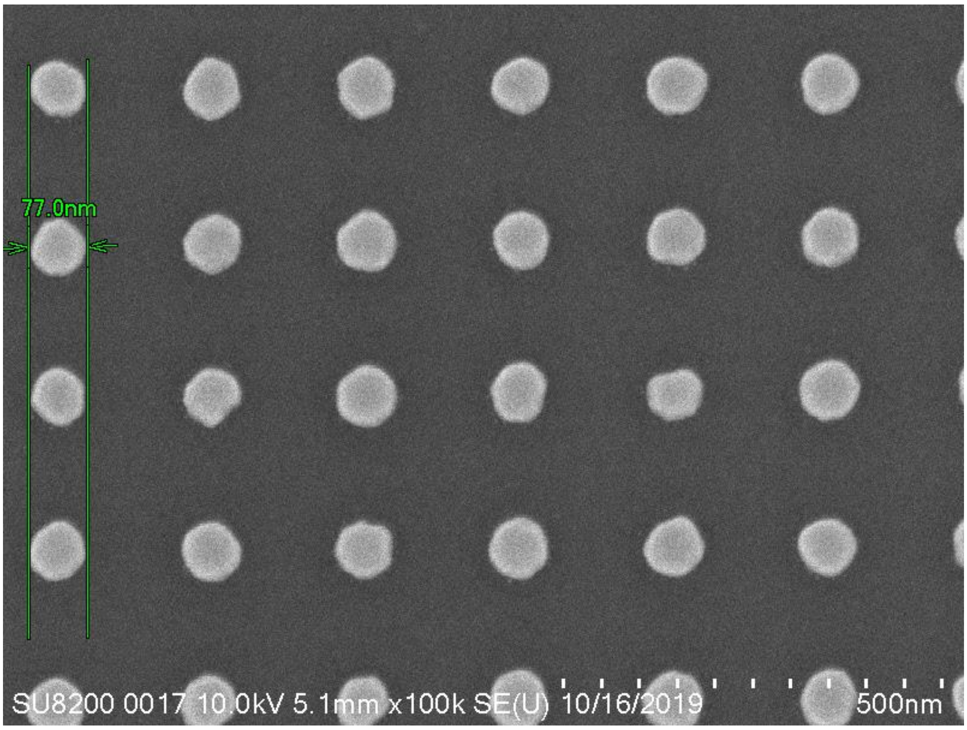

Fig. SI4. (a) SEM image of a fragment of the Au nanodisk array with a period of 200 nm
